# Is first pregnancy age associated with hypertension in the Chinese rural women population?

**DOI:** 10.3389/fpubh.2023.1120732

**Published:** 2023-04-17

**Authors:** Zhen Du, Xueyan Wu, Wei Liao, Ze Hu, Jing Yang, Xiaokang Dong, Hongfei Zhao, Xiaotian Liu, Chongjian Wang, Bing Zhao

**Affiliations:** ^1^Department of Obstetrics and Gynecology, The Third Affiliated Hospital of Zhengzhou University, Zhengzhou, Henan, China; ^2^Department of Epidemiology and Biostatistics, College of Public Health, Zhengzhou University, Zhengzhou, Henan, China

**Keywords:** first pregnancy age, hypertension, blood pressure indicators, rural population, women

## Abstract

**Introduction:**

The purpose of this study was to investigate the relationship between first pregnancy age and hypertension later in the life of women from Chinese rural areas.

**Methods:**

In total, 13,493 women were enrolled in the Henan Rural Cohort study. Logistic regression and linear regression were used to evaluate the association between first pregnancy age and hypertension and blood pressure indicators [including systolic blood pressure (SBP), diastolic blood pressure (DBP), and mean arterial pressure (MAP)]. The restricted cubic spline was used to examine the dose–response relationship between the first pregnancy age and hypertension or blood pressure indicators.

**Results:**

After adjusting for potential confounders, each 1-year increase in first pregnancy age was associated with a 0.221 mmHg increase in SBP values, a 0.153 mmHg increase in DBP values, and a 0.176 mmHg decrease in MAP values (all *P* < 0.05). The *β* of SBP, DBP, and MAP showed a trend of first increasing and then decreasing with increasing first pregnancy age and there was no statistical significance after first pregnancy age beyond 33 years on SBP, DBP, and MAP, respectively. A 1-year increment in first pregnancy age was associated with a 2.9% [OR (95% CI): 1.029 (1.010, 1.048)] higher odds of prevalent hypertension. The odds of hypertension increased sharply and then eventually leveled off with an increment of first pregnancy age after adjusting for potential confounders.

**Conclusion:**

First pregnancy age might increase the risk of hypertension later in life and might be an independent risk factor for hypertension in women.

## Introduction

Hypertension is a principal cause of the cardiovascular disease (CVD), which has become one of the most severe diseases in the world and China (1, 2). Nearly one-third of these deaths in 2019 were due to CVD. The leading risk factor is raised blood pressure (BP) or hypertension, which accounted for 10.8 million deaths (19.2% of all deaths in 2019) and 9.3% of disability-adjusted life-years lost globally (3). According to the latest data released by the “Report on Disease of Cardiovascular in China 2020,” the standardized prevalence of hypertension was 23.20% in China (4). Sex differences in the prevalence of hypertension are ubiquitous. A woman's lifetime undergoes many dynamic changes, including menarche, pregnancy, lactation, and menopause (5).

Pregnancy represents a unique challenge for the mother's body, especially the first pregnancy. Studies have shown that hormonal, immunological, and microbiological changes occur in the maternal body during pregnancy (6). In addition, this was particularly true for the first pregnancy, various potential factors that exist in the first pregnancy might have an impact on health later in life. Ozdemir et al. (7) showed that a higher first pregnancy age will increase the risk of osteoporosis. Moreover, first pregnancy age is associated with cancer (8), metabolic syndrome (9), and diabetes (10) in future. Therefore, issues related to first pregnancy age and future health risks are attracting increased attention. However, most of the studies are from foreign populations or urban areas in China. According to the different populations, the research results are different, and there are few studies in rural areas. China is a large agricultural country with severe aging of the rural population and limited medical resources. Focusing on the future health status of rural women will help reduce the burden of the country and enhance social harmony.

Thus, the purpose of this study was to investigate the relationship between first pregnancy age and hypertension later in the life of women from Chinese rural areas.

## Methods

### Study design and participants

A cross-sectional study was implemented from the baseline data of the Henan Rural Cohort Study launched in Henan from July 2015 to September 2017. More details of the cohort have been described in the previous study (11). In summary, this rural-based study incorporated a total of 39,259 participants (23,769 were women) aged 18–79 years, and a 93.7% response rate was reported (11). In this study, 13,749 female participants with complete information on first pregnancy age were included. The participants were excluded who were without the information on hypertension (*n* = 22) or first pregnancy age <18 years (*n* = 44) or suffering from cancer (*n* = 167). Finally, 13,493 female participants were included in our study. All the participants provided informed consent. The study was approved by the “Life Science Ethics Committee of Zhengzhou University.”

### Assessment of first pregnancy age

The first pregnancy age was calculated from the date of delivery of the first baby and the date of birth of the mother (12). The information was obtained by self-reporting using a standard questionnaire. Participants were asked an open question: “when did you have your first pregnancy?” Details about the questionnaire have been introduced elsewhere (13).

### Definition of hypertension

Hypertension was considered in patients who were with systolic blood pressure (SBP) of at least 140 mmHg or diastolic blood pressure (DBP) of at least 90 mmHg or self-reported hypertension diagnosed by a physician and patients who were taking current antihypertensive treatment during the last 2 weeks (14). A family history of hypertension was defined as at least one first-degree member with hypertension. Blood pressure was measured three times by electronic sphygmomanometer (Omron HEM-7071A, Japan) in the right arm in the sitting position after at least 5 min rest. There were 30 s intervals between the three measurements. Mean arterial pressure (MAP) was calculated using the formula, MAP = DBP+1/3(SBP − DBP). More detailed descriptions were previously published (15).

### Other covariate variables

Participants' demographic characteristics (age, marital status, educational level, and family per capita annual income), lifestyle behaviors (smoking status, drinking status, and physical activity), dietary, and reproductive factors (age at menarche, menopause status, age at menopause, parity information, use of oral contraceptive pills, breastfeeding, and gestational hypertension, gestational diabetes mellitus, and age at last birth) were collected by a standard questionnaire. Education levels were divided into three groups: elementary school or below, middle school, and high school or above. Family per capita annual income (RMB) was divided into four groups: ≤ 10,000, 10,001~, 20,001~, and 50,001~. Marital status was divided into single/widowed/separated/divorced and married/cohabiting. Smokers were defined as a person who smoked more than one cigarette per day in the past 6 months (16). Participants who consumed alcohol 12 or more times every year were viewed as drinkers (11). The Physical Activity Questionnaire (IPAQ 2001) was used to assess the levels of physical activity (17). Adequate intake of vegetables and fruits was defined as a person who consumed an average of more than 500 g of vegetables and fruits per day (18). High-fat diet was defined as a person who took an average of more than 75 g of livestock and poultry meat per day (19).

Height, weight, and blood pressure are measured by trained personnel according to standard instructions described elsewhere (20). Body mass index (BMI, kg/m^2^) was calculated as weight (kg) divided by the square of height (m). The details of the equipment for anthropometric and clinical examinations have been introduced elsewhere (21, 22).

### Statistical analysis

Categorical variables were presented as numbers (percentages), and the Chi-square test was used to compare the participants' baseline characteristics. Continuous variables were reported as mean ± standard deviation (SD), and the Student's *t*-test was used for comparative analysis. Five models were developed to assess associations of first pregnancy age and hypertension or blood pressure indicators (SBP, DBP, and MAP) by using the logistic regression model or linear regression. We produced five models as follows: Model 1: adjusted for age, marital status, education level, family per capita yearly income, smoking, alcohol consumption, physical activity, adequate intake of vegetables and fruits, high-fat diet, family history of hypertension, and BMI; Model 2: adjusted as in model 1 plus age at menarche, menopause status, breastfeeding, and use of oral contraceptive pills; Model 3: adjusted as in model 2 plus gestational hypertension and gestational diabetes mellitus; Model 4: adjusted as in model 3 plus parity; Model 5: adjusted as in model 4 plus age at last birth. We examined the dose–response relationship between the first pregnancy age and hypertension or blood pressure indicators using the restricted cubic spline analysis. We furthermore performed subgroup analyses by several factors: age, education level, averaged yearly income, adequate intake of vegetables and fruits, high-fat diet, physical activity, age at menarche, menopause status, and parity ≤ 2. The interactions were performed to test the effect modification in subgroup analyses by a generalized linear model. In addition, according to the outcome of the first pregnancy, we further assessed associations of first pregnancy age and hypertension or blood pressure indicators (SBP, DBP, and MAP) by using a logistic regression model or linear regression. Considering that the associations of first pregnancy age with hypertension or blood pressure indicators might be attributable to their outliers, we conducted a sensitivity analysis to examine the linearity of associations of age at first pregnancy which was fixed at 2.5th−97.5th percentile ranges and hypertension or blood pressure indicators. In addition, to exclude the effect of taking antihypertensive drugs on blood pressure indices, we further excluded the participants who used antihypertensive medicine and performed sensitivity analysis. Moreover, we analyzed the association with hypertension using age at first birth rather than age at first pregnancy to test the credibility of the results.

All data were analyzed by SPSS software V.26.0 and R version 4.0.0. Statistical significance was set to a *p*-value of <0.05 at two tails.

## Results

### Basic characteristics of the study population

Table 1 shows the main demographic characteristics of 13,493 female participants aged from 19 to 79 years according to hypertension status. The mean ± SD age at recruitment was 54.99 ± 12.14 years, and the mean ± SD age of first pregnancy was 23.57 ± 2.47 years. Compared to those without hypertension, those with hypertension were likely to be older, with higher levels of BMI, first pregnancy age, age at menarche, age at menopause, parity, SBP, DBP, and MAP (all *P* < 005). In addition, those with hypertension were likely to be unmarried/divorced/widowed and postmenopausal; have lower levels of education, yearly income, and physical activity; have a not-so-high-fat diet and enough vegetables and fruits; and report a family history of hypertension. Distributions of the selected variables were statistically significantly different between hypertension and normotensive individuals groups (all *P* < 0.05), except for the distribution of smoking status (*P* = 0.796) and gestational hypertension (*P* = 0.530).

**Table 1:** Demographic characteristics of participants according to hypertension status.

**Variables**	**Total**	**Normotensive**	**Hypertension**	***P^*a*^***
*N* (%)	13,493 (100)	8,861 (65.67)	4,632 (34.33)	
Age (year)	54.99 ± 12.14	51.65 ± 12.09	61.38 ± 9.35	<0.001
**Marital status**				<0.001
Married/cohabitation	12,223 (90.59)	8,254 (93.15)	3,969 (85.69)	
Unmarried/divorced/ widowed	1,270 (9.41)	607 (6.85)	663 (14.31)	
**Educational level**				<0.001
Elementary school or below	6,883 (51.01)	3,864 (43.61)	3,019 (65.18)	
Middle school	4,646 (34.43)	3,423 (38.63)	1,223 (26.40)	
High school or above	1,964 (14.56)	1,574 (17.76)	390 (8.42)	
**Income (RMB)** ^b^				<0.001
≤ 10,000	3,009 (22.33)	1,748 (19.74)	1,261 (27.30)	
10,001~	2,382 (17.68)	1,525 (17.22)	857 (18.55)	
20,001~	4,714 (34.98)	3,202 (36.16)	1,512 (32.73)	
50,001~	3,370 (25.01)	2,381 (26.89)	989 (21.41)	
**Physical activity**				<0.001
Low	4,378 (32.45)	2,679 (30.23)	1,699 (36.68)	
Moderate	5,215 (38.65)	3,646 (41.15)	1,569 (33.87)	
High	3,900 (28.90)	2,536 (28.62)	1,364 (29.45)	
Current regular smokers	40 (0.30)	25 (0.28)	15 (0.32)	0.796
Current regular drinking	257 (1.90)	199 (2.25)	58 (1.25)	<0.001
Vegetable/fruit (yes)^c^	5,778 (42.82)	4,052 (45.73)	1,726 (37.26)	<0.001
High fat diet (yes)	1,627 (12.06)	1,239 (13.98)	388 (8.38)	<0.001
BMI (kg/m^2^)	25.16 ± 3.64	24.54 ± 3.42	26.35 ± 3.75	<0.001
First pregnancy age (years)	23.57 ± 2.47	23.53 ± 2.43	23.66 ± 2.54	0.003
Age at last birth (years)	29.89 ± 4.43	29.66 ± 4.44	30.35 ± 4.38	<0.001
Age at menarche (years)	15.74 ± 2.16	15.49 ± 2.10	16.21 ± 2.20	0.004
Age at menopause (years)	48.97 ± 4.81	48.84 ± 4.77	49.13 ± 4.85	<0.001
**Menopause status**				<0.001
Premenopausal	4,682 (34.70)	4,024 (45.41)	658 (14.21)	
Postmenopausal	8,811 (65.30)	4,837 (54.59)	3,974 (85.79)	
Use of oral contraceptive pills	356 (2.64)	281 (3.17)	75 (1.62)	<0.001
Parity	2.53 ± 1.02	2.37 ± 0.93	2.84 ± 1.12	<0.001
Gestational hypertension	105 (0.78)	72 (0.81)	33 (0.71)	0.530
Gestational diabetes mellitus	17 (0.13)	15 (0.17)	2 (0.04)	0.050
Family history of hypertension	2,689 (19.93)	1,432 (16.16)	1,257 (27.14)	<0.001
SBP (mmHg)	126.44 ± 21.11	115.15 ± 12.24	148.05 ± 17.36	<0.001
DBP (mmHg)	77.22 ± 11.43	72.05 ± 8.03	87.11 ± 10.39	<0.001
MAP (mmHg)	93.62 ± 13.85	86.41 ± 8.68	107.42 ± 11.16	<0.001

a Chi-square test or t-tests;

b per capita annual income (RMB);

c Adequate intake of vegetables and fruits (yes). BMI, body mass index, weight (kg)/height (m)^2^; SBP, systolic blood pressure; DBP, diastolic blood pressure; MAP, mean arterial pressure. Values are means and standard deviation for continuous variables, and numbers and percentages for categorical variables.

### Association of first pregnancy age with blood pressure indicators

**Figure 1:**
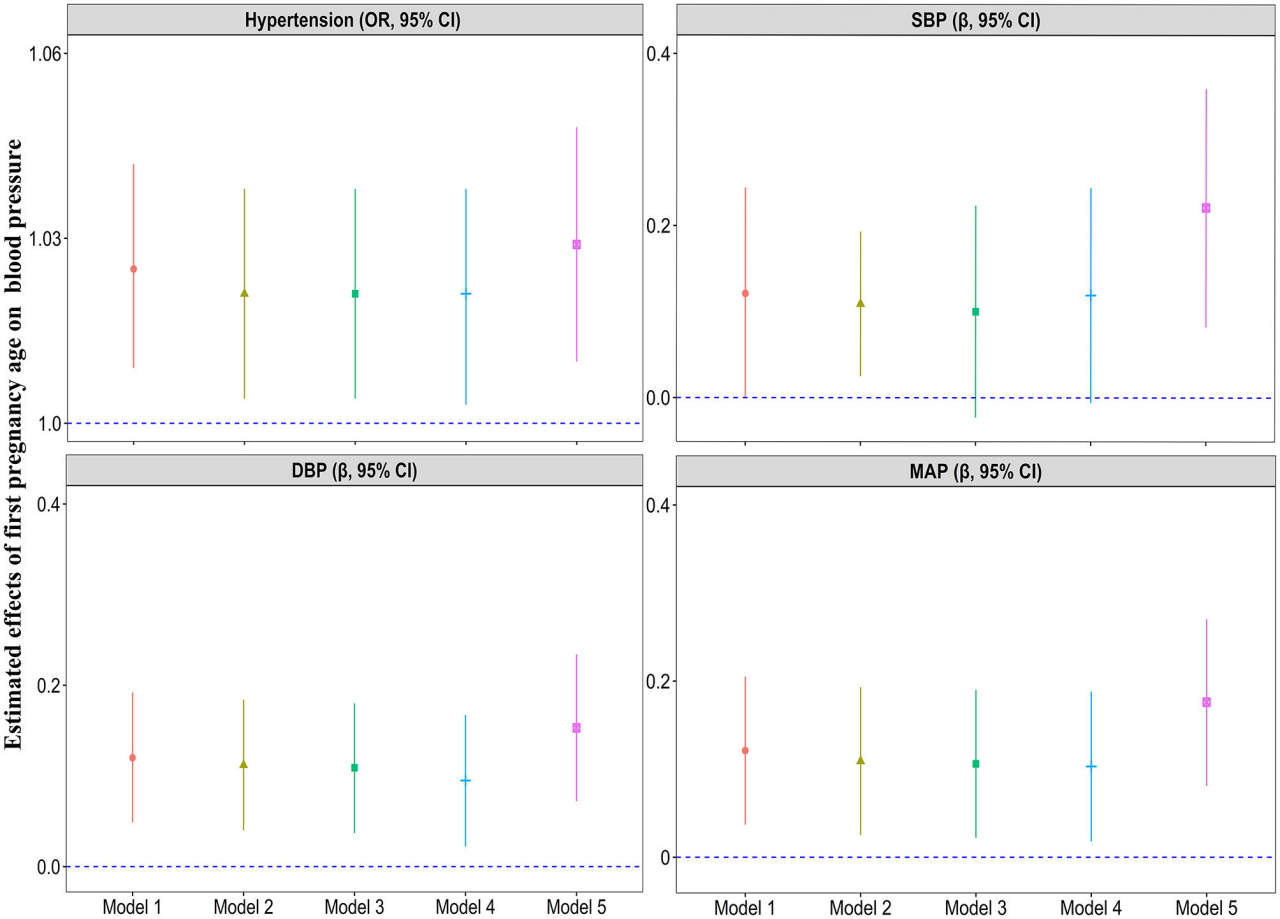
Multivariable analysis of first pregnancy age with hypertension, SBP, DBP, and MAP. Model 1: adjusted for age, marital status, education level, family per capita yearly income, smoking, alcohol consumption, physical activity, adequate intake of vegetables and fruits, high-fat diet, family history of hypertension, and BMI; Model 2: adjusted as in model 1 plus age at menarche, menopause status, breastfeeding, and use of oral contraceptive pills; Model 3: adjusted as in model 2 plus gestational hypertension and gestational diabetes mellitus; Model 4: adjusted as in model 3 plus parity; Model 5: adjusted as in model 4 plus age at last birth.

**Figure 2:**
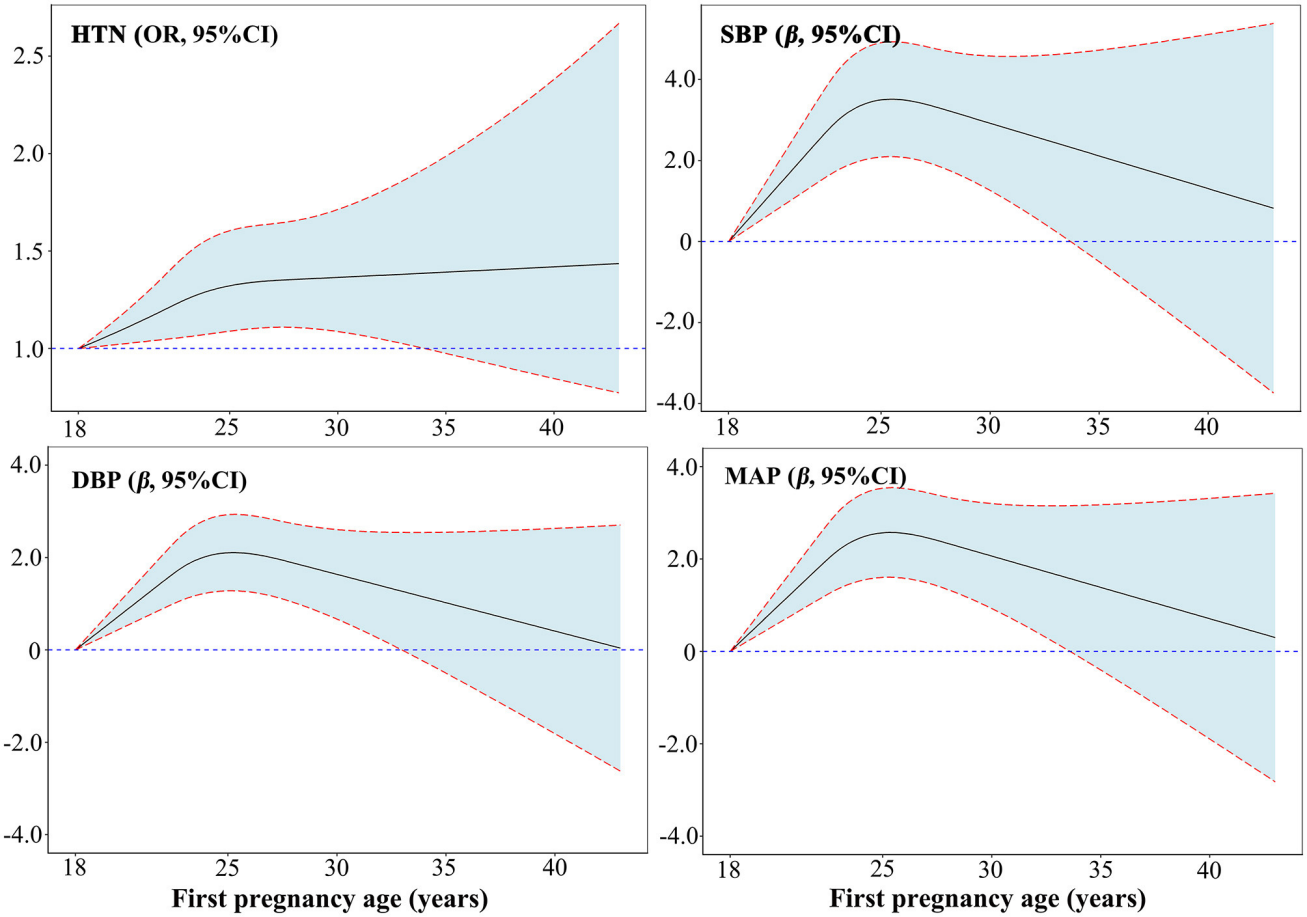
Dose–responsive relationship of first pregnancy age with hypertension, SBP, DBP, and MAP from restricted cubic splines. Adjusted for age, marital status, education level, family per capita yearly income, smoking, alcohol consumption, physical activity, adequate intake of vegetables and fruits, high-fat diet, family history of hypertension, BMI, age at menarche, menopause status, breastfeeding, use of oral contraceptive pills, gestational hypertension, gestational diabetes mellitus, parity, and age at last birth.

Figure 1 and Supplementary Table S1 present the results of multivariable analysis of first pregnancy age and blood pressure indicators (SBP, DBP, and MAP). Each 1-year increase in first pregnancy age was associated with a 0.109 mmHg increase in SBP values (95% CI: 0.025, 0.193), a 0.112 mmHg increase in DBP values (95% CI: 0.040, 0.184), and a 0.109 mmHg decrease in MAP values (95% CI: 0.025, 0.193) in model 2 adjusting for age, marital status, education level, family per capita yearly income, smoking, alcohol consumption, physical activity, adequate intake of vegetables and fruits, high-fat diet, family history of hypertension, BMI, age at menarche, menopause status, breastfeeding, and use of oral contraceptive pills (all *P* < 0.05). A significant positive association between first pregnancy age, DBP, and MAP was observed in Model 3 (further adjustment of gestational hypertension and gestational diabetes mellitus) and Model 4 (further adjustment of parity). The effect of first pregnancy age on SBP [*β* (95% CI): 0.221 (0.082, 0.359)], DBP [*β* (95% CI): 0.153 (0.072, 0.234)], and MAP [*β* (95% CI): 0.176 (0.081, 0.270)] was stronger after adjusting for age at last birth (Model 5) and remained statistically significant. However, no association was observed between first pregnancy age and SBP in Model 3 (further adjustment of gestational hypertension and gestational diabetes mellitus) and Model 4 (further adjustment of parity). To observe the trend between first pregnancy age and SBP, DBP, and MAP, multivariable restricted cubic regression splines were conducted (Figure 2). The spline analysis showed a significant non-linear relationship between first pregnancy age and SBP (*P-*overall association < 0.001; *P*-non-linear association = 0.001), DBP (*P-*overall association < 0.001; *P-*non-linear association < 0.001), and MAP (*P-*overall association < 0.011; *P*-non-linear association < 0.001). The *β* of SBP, DBP, and MAP showed a trend of first increasing and then leveling off or decreasing with increasing first pregnancy age and there was no statistical significance after first pregnancy age beyond 33 years on SBP, DBP, and MAP, respectively.

### Association of first pregnancy age with and hypertension

The results of the multivariable analysis of first pregnancy age and hypertension are shown in Figure 1 and Supplementary Table S1. A 1-year increment in first pregnancy age was associated with a 2.1% (95% CI: 1.004, 1.038) higher odds of prevalent hypertension in Model 2. After further adjusting for confounding factors including gestational hypertension and gestational diabetes mellitus (Model 3), parity (Model 4), and age at last birth (Model 5), the associations were still significant and the magnitude of associations was slightly decreased in Model 5 [OR (95% CI): 1.029 (1.010, 1.048)]. Moreover, age at first pregnancy was classified as a categorical variable (18–21, 22, 23, 24, 25, 26, and ≥27 years) according to a seven-point scale, with the 18–21 years category as the reference category (Supplementary Table S2). Age at first pregnancy was positively associated with risk of hypertension with adjusted OR (95% CI) of 1.000, 1.235 (1.067, 1.431), 1.223 (1.062, 1.408), 1.194 (1.037, 1.374), 1.212 (1.042, 1.410), 1.273 (1.063, 1.524), and 1.234 (1.038, 1.467) for those with age at first pregnancy 18–21 (reference), 22, 23, 24, 25, 26, and ≥27 years, respectively (*P* trend = 0.021). Figure 2 displays the dose–responsive relationship between first pregnancy age and hypertension from restricted cubic splines with three knots placed at the 5th, 50th, and 95th. The spline analysis showed a significant linear relationship between first pregnancy age and hypertension (*P-*overall association = 0.011; *P*-non-linear association = 0.169). The odds of hypertension increased sharply and then eventually leveled off with an increment of first pregnancy age after adjusting for potential confounders. However, there was no statistical significance after the first pregnancy age beyond 34 years.

### Subgroup analyses and sensitivity analyses

**Figure 3:**
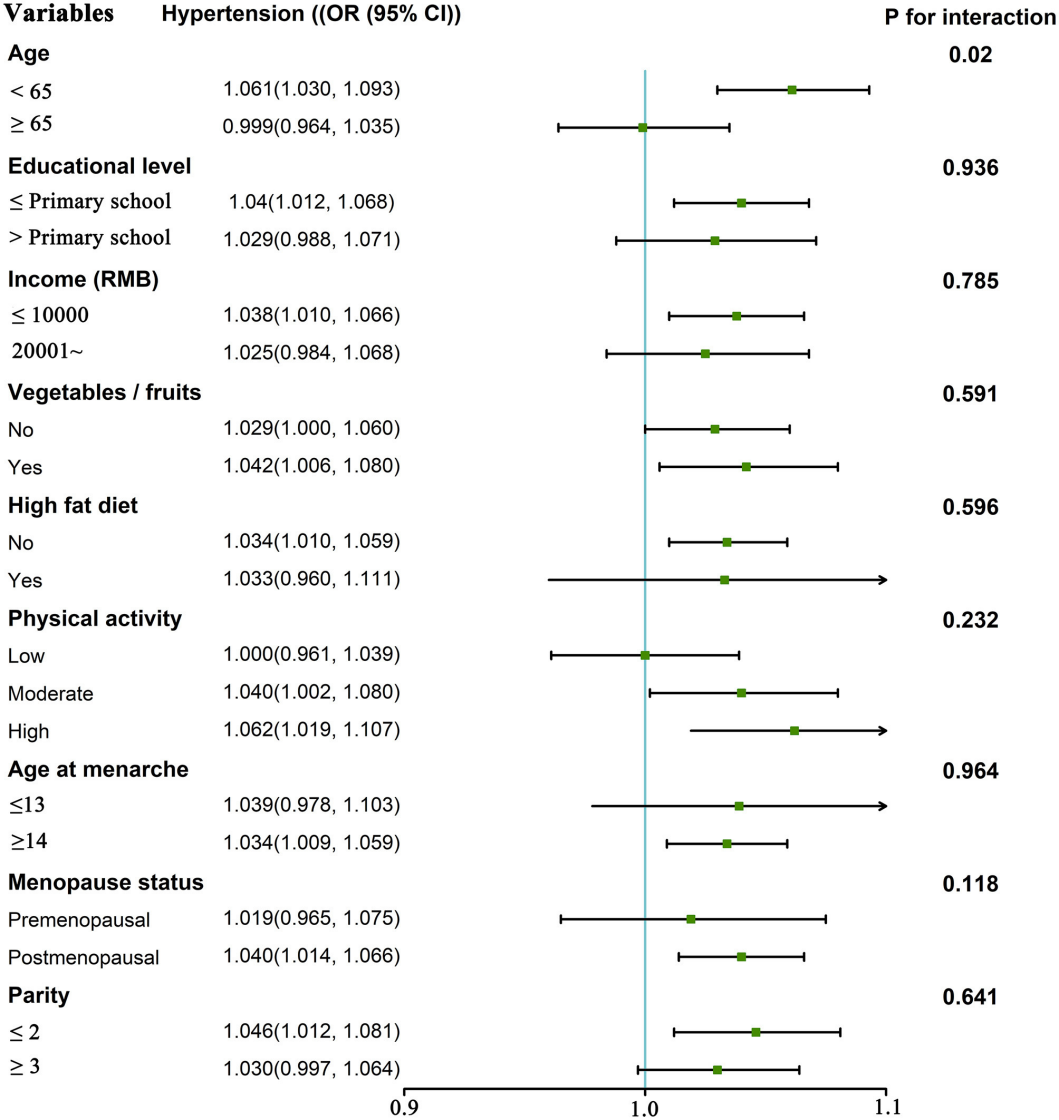
The odds ratio of hypertension (and 95% CI) associated with first pregnancy age according to potential modifiers. Adjusted for age, marital status, education level, family per capita yearly income, smoking, alcohol consumption, physical activity, adequate intake of vegetables and fruits, high-fat diet, family history of hypertension, BMI, age at menarche, menopause status, breastfeeding, use of oral contraceptive pills, gestational hypertension, gestational diabetes mellitus, parity, and age at last birth (unless stratified by the respective factor).

The results of subgroup analyses of first pregnancy age and hypertension and blood pressure indicators are revealed in Supplementary Table S3 and Figure 3. After stratification by age, we found that the first pregnancy age was significantly associated with hypertension among younger female participants (<65 years). Statistically significant interactions were only detected between first pregnancy age and age (*P* interaction = 0.029). For the associations between first pregnancy age and SBP, first pregnancy age was significantly associated with higher SBP levels in subgroups including age <65 years, education levels ≤ primary school, no high-fat diet, high physical activity, age at menarche ≥14 years, and parity ≤ 2 subgroups. First pregnancy age was significantly associated with higher DBP levels in subgroups including the age of 65 years, education levels ≤ Primary school, income <10,000 Yuan, adequate intake of vegetables and fruits, no high-fat diet, high-fat diet, age at menarche ≥14 years, postmenopausal, and parity ≥2. First pregnancy age was significantly associated with higher MAP levels in subgroups including age <65 years, education levels ≤ Primary school, income <10,000 Yuan, adequate intake of vegetables and fruits, no high-fat diet, high-fat diet, age at menarche ≥14 years, postmenopausal, and parity ≤ 2. In addition, statistically significant interactions were only detected between first pregnancy age and age on SBP (*P*_interaction_ = 0.029). Furthermore, we did not observe significant interactions between first pregnancy age and age, education levels, economic conditions, adequate intake of vegetables and fruits, high-fat diet, physical activity, age at menarche, menopause status, or parity on SBP, DBP, and MAP (all *P*_interaction_ > 0.05). In addition, we divided the group into two groups based on the first pregnancy outcome: the first pregnancy with the delivery group and the first pregnancy with the abortion group (Supplementary Tables S4). A meaningful positive association between age at first pregnancy and both risks of hypertension and blood pressure indicators (SBP, DBP, and MAP) was found among women in the first pregnancy with normal delivery (all *P* < 0.05). No association was found between age at first pregnancy and both risk of hypertension and blood pressure indicators among women in the first pregnancy miscarriage. However, there were no significant between-group differences (all *P* ≥ 0.05).

The results of the sensitivity analyses are shown in Supplementary Table S5. The estimated effects of the associations between first pregnancy age and hypertension, SBP, DBP, and MAP did not appreciably change the result after fixing the levels of first pregnancy age at the 2.5th and 97.5th ranges. In addition, a sensitivity analysis excluding the participants who used anti-hypertensive medicine yielded similar results (Supplementary Tables S6). Moreover, when we analyzed the association with hypertension using age at first birth rather than age at first pregnancy, the results were robust (Supplementary Table S7).

## Discussion

In this study, we explored a higher likelihood of the relationship between first pregnancy age and hypertension later in life in depth. Our study showed that elevated first pregnancy age was associated with an increased risk of hypertension and increased levels of blood pressure parameters after adjusting for potential confounders including age, marital status, education level, family per capita yearly income, smoking, alcohol consumption, physical activity, adequate intake of vegetables and fruits, high-fat diet, family history of hypertension, BMI, age at menarche, menopause status, breastfeeding, use of oral contraceptive pills, gestational hypertension gestational diabetes mellitus, parity, and age at last birth. A 1-year increment in first pregnancy age was associated with a 2.9% higher risk of hypertension after adjusting for confounding factors. However, there was no statistical significance after the first pregnancy age beyond 34 years.

Our study suggested that the risk of hypertension increased with the increasing age of the first pregnancy after adjusting for potential confounders in rural Chinese women. Previous studies have shown that reproductive factors were associated with many unfavorable outcomes, such as hypertension (23), metabolic syndrome (24), and cardiovascular disease (25). It is important to identify individuals at high risk for unfavorable outcomes in women attributed to reproductive factors, which would allow them to benefit from early intervention. Our results indicated that age at first pregnancy might be a significant predictor of a woman's health as well. However, there was inconsistency regarding the association between age at first pregnancy and adverse health outcomes in later life. A study showed that women who have a first child at an earlier age were more likely to have cardiovascular disease (26). An observational cohort study of healthy aging in Australia found that women who were older when they gave their first birth had lower odds of treatment for high blood pressure compared with women who were younger when they gave birth to their first child (27). The age at first birth was used according to similar studies. Moreover, when we analyzed the association with hypertension using age at first birth rather than age at first pregnancy, the results were similar. We have demonstrated the plausibility of this association that later first pregnancy age might increase the risk of hypertension by adjusting the possible potential factors and stratifying the analysis as much as possible. Furthermore, a previous study showed that one in five rural Chinese postpartum women with a history of gestational diabetes mellitus were found to have elevated blood pressure (28). The likelihood of early ischemic heart disease and stroke is higher in women with a history of hypertension during pregnancy (29, 30). To exclude the effect of gestational diabetes mellitus and gestational hypertension, we further conducted a sensitivity analysis to examine the associations between age at first pregnancy and hypertension or blood pressure indicators after excluding gestational hypertension and gestational diabetes mellitus, the estimated effects of the associations between first pregnancy age and hypertension, SBP, DBP, and MAP did not appreciably change the result after excluding gestational hypertension and gestational diabetes mellitus, and the result further demonstrated the stability and feasibility of our results. A later age of first pregnancy might not only lead to some serious adverse reproductive outcomes (31) but also increase the risk of hypertension later in life. Therefore, an appropriate age at first pregnancy strategy is likely to help provide preventive strategies and improve the future health of women. Before the present study, there was no study carried out in China to investigate the impact of age at first pregnancy on the risk of hypertension, which was useful for the screening and intervention of hypertension among Chinese rural women. However, the conflicting findings from the limited body of previous studies were partly accounted for by the variations in study design, population, and sample size.

Notably, the association between first pregnancy age and hypertension was even stronger in younger women (age < 65 years), which reinforced the idea that age at first pregnancy is an independent influence on hypertension. In addition, we divided the group into two groups based on the first pregnancy outcome: the first pregnancy with the delivery group and the first pregnancy with the abortion group. A meaningful positive association between age at first pregnancy and the risk of hypertension and the levels of SBP, DBP, and MAP was found among women in the first pregnancy with normal delivery. No association was found between age at first pregnancy and both risk of hypertension and blood pressure indicators among women in the first pregnancy miscarriage. However, further analysis of subgroup differences was carried out and no differences were found. Therefore, we could not obtain similar results after stratified analysis that later first pregnancy age might increase the risk of hypertension according to the outcome of the first pregnancy. In addition, the number of women included in this study who had first pregnancy and miscarriage was small. Further exploration and validation in large samples and prospective cohort studies are still needed.

### Strengths and limitations

Our study is based on the relatively large sample size of the rural population in China. Standardized investigation tools, training, and on-site implementation, as well as adjustments of a wide range of potential confounding factors, ensure the reliability of the analysis. Furthermore, this is the first description of the association of age at first pregnancy with hypertension for women in rural areas of China. Nevertheless, several limitations should also be considered in the current analysis. First, these findings came from a cross-sectional study, rather than a prospective cohort design, and thus do not accurately describe causality. Second, the participants are mainly rural women in China. Whether the observed association could be applied to other ethnic groups and areas warrants further investigation. Third, although we have comprehensively considered typical risk factors and potential confounders, residual confounding is inevitable owing to the observational study design.

## Conclusion

Our study showed that the first pregnancy age might increase the risk of hypertension later in the life of women from Chinese rural areas. First pregnancy age might be an independent risk factor for hypertension in women. However, further prospective studies are anticipated to assess the causality and the specific mechanism of the association.

## Data availability statement

The original contributions presented in the study are included in the article/Supplementary material, further inquiries can be directed to the corresponding authors.

## Ethics statement

The studies involving human participants were reviewed and approved by the Henan Rural Cohort Study has been registered at Chinese Clinical Trial Register (Registration Number: ChiCTR-OOC-15006699). Date of registration: 2015-07-06. http://www.chictr.org.cn/showproj.aspx?proj=11375. The patients/participants provided their written informed consent to participate in this study.

## Author contributions

ZD: data curation, formal analysis, visualization, and writing-original draft. XW: investigation, data curation, methodology, formal analysis, visualization, and writing-original draft. WL and ZH: investigation, validation, and writing-review and editing. JY, XD, HZ, and XL: investigation and writing-review and editing. CW: conceptualization, methodology, investigation, validation, supervision, funding acquisition, project administration, and writing-original draft. BZ: investigation, data curation, and writing-review and editing. All authors contributed to the article and approved the submitted version.
