# Supplementary material for: Is first pregnancy age associated with hypertension in the Chinese rural women population?
Source: Front Public Health. 2023 Apr 17;11:1120732. doi: 10.3389/fpubh.2023.1120732 (PMC10150638; doi:10.3389/fpubh.2023.1120732)
Supplement: Supplementary file 1 [file Data_Sheet_1.docx]

**Supplementary Table 1.** Multivariable analysis of first pregnancy age (per 1 year increment) and current hypertension, SBP, DBP, and MAP.

| **Models** | **Hypertension** | **SBP** | **DBP** | **MAP** |
| --- | --- | --- | --- | --- |
| Model 1 | 1.025(1.009, 1.042)* | 0.121(-0.001, 0.244) | 0.120(0.049, 0.192)* | 0.121(0.037, 0.205)* |
| Model 2 | 1.021(1.004, 1.038)* | 0.109(0.025, 0.193)* | 0.112(0.040, 0.184)* | 0.109(0.025, 0.193)* |
| Model 3 | 1.021(1.004, 1.038)* | 0.100(-0.023, 0.223) | 0.109(0.037, 0.180)* | 0.106(0.022, 0.190)* |
| Model 4 | 1.021(1.003, 1.038)* | 0.119(-0.006, 0.244) | 0.095(0.022, 0.167)* | 0.103(0.018, 0.188)* |
| Model 5 | 1.029(1.010, 1.048)* | 0.221(0.082, 0.359)* | 0.153(0.072, 0.234)* | 0.176(0.081, 0.270)* |

Data are OR (95% CI) or *β* (95% CI). ^*^, *P* < 0.05.

SBP, systolic blood pressure; DBP, diastolic blood pressure; MAP, mean arterial pressure.

Model 1: adjusted for age, marital status, education level, family per capita yearly income, smoking, alcohol consumption, physical activity, adequate vegetables and fruits intake, high fat diet, family history of hypertension, and BMI; Model 2: adjusted as in model 1 plus age at menarche, menopause status, breast feeding, and use of oral contraceptive pills; Model 3: adjusted as in model 2 plus gestational hypertension and gestational diabetes mellitus; Model 4: adjusted as in model 3 plus parity; Model 5: adjusted as in model 4 plus age at last birth.

**Supplementary Table 2.** ORs (95% *CI*s) for hypertension according to age at first pregnancy groups

| **Age at first pregnancy**  **(years)** | **Hypertension**  **(OR, 95% CI)** | ***P*** |
| --- | --- | --- |
| 18-21 | Reference |  |
| 22 | 1.235(1.067, 1.431) | 0.005 |
| 23 | 1.223(1.062, 1.408) | 0.005 |
| 24 | 1.194(1.037, 1.374) | 0.014 |
| 25 | 1.212(1.042, 1.410) | 0.013 |
| 26 | 1.273(1.063, 1.524) | 0.009 |
| ≥27 | 1.234(1.038, 1.467) | 0.017 |
| P trend | 1.029(1.004, 1.054) | 0.021 |

Note: Results were adjusted for age, marital status, education level, family per capita yearly income, smoking, alcohol consumption, physical activity, adequate vegetables and fruits intake, high fat diet, family history of hypertension, BMI, age at menarche, menopause status, breast feeding, use of oral contraceptive pills, gestational hypertension, gestational diabetes mellitus, parity, and age at last birth.

**Supplementary Table 3.** Associations between first pregnancy age and SBP, DBP, and MAP stratified by potential modifiers.

| **Variables** | **SBP** | |  | **DBP** | |  | **MAP** | |
| --- | --- | --- | --- | --- | --- | --- | --- | --- |
|  | ***β* (95% CI)** | ***P*** ^a^ |  | ***β* (95% CI)** | ***P*** ^a^ |  | ***β* (95% CI)** | ***P*** ^a^ |
| **Age** |  | 0.029 |  |  | 0.558 |  |  | 0.45 |
| < 65 | 0.448(0.241, 0.656)* |  |  | 0.117(-0.006, 0.240) |  |  | 0.227(0.083, 0.371)* |  |
| ≥ 65 | -0.126(-0.473, 0.222) |  |  | 0.185(0.005, 0.366)* |  |  | 0.082(-0.139, 0.302) |  |
| **Educational level** |  | 0.352 |  |  | 0.125 |  |  | 0.185 |
| ≤ Primary school | 0.296(0.056, 0.536)* |  |  | 0.223(0.093, 0.353)* |  |  | 0.247(0.090, 0.404)* |  |
| > Primary school | 0.128(-0.132, 0.388) |  |  | -0.004(-0.162, 0.153) |  |  | 0.04(-0.142, 0.222) |  |
| **Income (RMB)^b^** |  | 0.799 |  |  | 0.629 |  |  | 0.884 |
| ≤ 10000 | 0.192(-0.033, 0.416) |  |  | 0.138(0.013, 0.263)* |  |  | 0.156(0.006, 0.305)* |  |
| 20001~ | 0.251(-0.032, 0.535) |  |  | 0.065(-0.101, 0.232) |  |  | 0.127(-0.067, 0.322) |  |
| **Vegetables and fruits^c^** | | 0.876 |  |  | 0.606 |  |  | 0.713 |
| No | 0.235(-0.003, 0.472) |  |  | 0.162(0.030, 0.294)* |  |  | 0.186(0.029, 0.344)* |  |
| Yes | 0.223(-0.037, 0.482) |  |  | 0.072(-0.08, 0.223) |  |  | 0.122(-0.056, 0.300) |  |
| **High fat diet** |  | 0.778 |  |  | 0.747 |  |  | 0.748 |
| No | 0.166(0.039, 0.293)* |  |  | 0.130(0.023, 0.237)* |  |  | 0.166(0.039, 0.293)* |  |
| Yes | 0.040(-0.279, 0.359) |  |  | 0.026(-0.251, 0.304) |  |  | 0.040(-0.279, 0.359) |  |
| **Physical activity** |  | 0.767 |  |  | 0.771 |  |  | 0.767 |
| Low | -0.104(-0.417, 0.209) |  |  | 0.074(-0.101, 0.249) |  |  | 0.015(-0.194, 0.223) |  |
| Moderate | 0.287(0.011, 0.564)* |  |  | 0.113(-0.046, 0.272) |  |  | 0.171(-0.016, 0.358) |  |
| High | 0.501(0.169, 0.833)* |  |  | 0.172(-0.018, 0.361) |  |  | 0.282(0.057, 0.506)* |  |
| **Age at menarche** |  | 0.356 |  |  | 0.361 |  |  | 0.331 |
| ≤13 | 0.088(-0.294, 0.469) |  |  | -0.02(-0.256, 0.215) |  |  | 0.016(-0.254, 0.286) |  |
| ≥14 | 0.255(0.059, 0.451)* |  |  | 0.151(0.041, 0.261)* |  |  | 0.186(0.055, 0.317)* |  |
| **Menopause status** |  | 0.283 |  |  | 0.237 |  |  | 0.283 |
| Premenopausal | 0.253(-0.012, 0.517) |  |  | 0.083(-0.089, 0.255) |  |  | 0.139(-0.055, 0.334) |  |
| Postmenopausal | 0.215(-0.009, 0.440) |  |  | 0.145(0.023, 0.268)* |  |  | 0.169(0.022, 0.316)* |  |
| **Parity** |  | 0.576 |  |  | 0.304 |  |  | 0.763 |
| ≤ 2 | 0.348(0.123, 0.573)* |  |  | 0.086(-0.047, 0.219) |  |  | 0.173(0.017, 0.329)* |  |
| ≥ 3 | 0.141(-0.153, 0.435) |  |  | 0.204(0.045, 0.363)* |  |  | 0.183(-0.008, 0.374) |  |

^a^, *P* for interaction; ^b^, per capita annual income (RMB); ^c^, Adequate vegetables and fruits intake (yes); ^*^, *P* < 0.05.

SBP, systolic blood pressure; DBP, diastolic blood pressure; MAP, mean arterial pressure.

Note: Results were adjusted for age, marital status, education level, family per capita yearly income, smoking, alcohol consumption, physical activity, adequate vegetables and fruits intake, high fat diet, family history of hypertension, BMI, age at menarche, menopause status, breast feeding, use of oral contraceptive pills, gestational hypertension, gestational diabetes mellitus, parity, and age at last birth (unless stratified by the respective factor).

**Supplementary Table** 4. Associations between age at first pregnancy and hypertension, SBP, DBP, and MAP according to the outcome of first pregnancy.

| **First pregnancy age sub-group** | **OR / *β* (95% CI)** | ***P*** | ***P ^a^*** |
| --- | --- | --- | --- |
| **Hypertension** |  |  | >0.05 |
| First pregnancy with delivery | 1.030(1.010, 1.049) | 0.003 |  |
| First pregnancy with abortion | 0.958(0.847, 1.084) | 0.496 |  |
| **SBP** |  |  | >0.05 |
| First pregnancy with delivery | 0.218(0.077, 0.360) | 0.003 |  |
| First pregnancy with abortion | 0.007(-0.661, 0.674) | 0.985 |  |
| **DBP** |  |  | >0.05 |
| First pregnancy with delivery | 0.155(0.072, 0.237) | <0.001 |  |
| First pregnancy with abortion | -0.055(-0.486, 0.377) | 0.804 |  |
| **MAP** |  |  | >0.05 |
| First pregnancy with delivery | 0.176(0.079, 0.273) | <0.001 |  |
| First pregnancy with abortion | -0.034(-0.522, 0.454) | 0.891 |  |

^a^, *P* for difference.

SBP, systolic blood pressure; DBP, diastolic blood pressure; MAP, mean arterial pressure.

Note: Results were adjusted for age, marital status, education level, family per capita yearly income, smoking, alcohol consumption, physical activity, adequate vegetables and fruits intake, high fat diet, family history of hypertension, BMI, age at menarche, menopause status, breast feeding, use of oral contraceptive pills, gestational hypertension, gestational diabetes mellitus, parity, and age at last birth.

**Supplementary Table 5.** Sensitivities analyses of the association between first pregnancy age which were fixed at 2.5 th - 97.5 th percentile ranges and hypertension, SBP, DBP, and MAP.

| **Models** | **Hypertension** | **SBP** | **DBP** | **MAP** |
| --- | --- | --- | --- | --- |
| Model 1 | 1.026(1.006, 1.045)* | 0.063(-0.083, 0.209) | 0.056(-0.032, 0.144) | 0.058(-0.044, 0.160) |
| Model 2 | 1.020(1.001, 1.040)* | 0.051(-0.096, 0.197) | 0.047(-0.041, 0.135) | 0.048(-0.054, 0.151) |
| Model 3 | 1.020(1.000, 1.040)* | 0.045(-0.101, 0.192) | 0.043(-0.045, 0.131) | 0.044(-0.058, 0.146) |
| Model 4 | 1.020(1.001, 1.040)* | 0.067(-0.081, 0.214) | 0.029(-0.060, 0.119) | 0.042(-0.062, 0.145) |
| Model 5 | 1.028(1.006, 1.050)* | 0.204(0.043, 0.365)* | 0.115(0.018, 0.212)* | 0.145(0.032, 0.257)* |

Data are OR (95% CI) or *β* (95% CI). ^*^, *P* < 0.05.

SBP, systolic blood pressure; DBP, diastolic blood pressure; MAP, mean arterial pressure.

Model 1: adjusted for age, marital status, education level, family per capita yearly income, smoking, alcohol consumption, physical activity, adequate vegetables and fruits intake, high fat diet, family history of hypertension, and BMI; Model 2: adjusted as in model 1 plus age at menarche, menopause status, breast feeding, and use of oral contraceptive pills; Model 3: adjusted as in model 2 plus gestational hypertension and gestational diabetes mellitus; Model 4: adjusted as in model 3 plus parity; Model 5: adjusted as in model 4 plus age at last birth.

**Supplementary Table 6.** Multivariable analysis of first pregnancy age (per 1 year increment) and SBP, DBP, and MAP by excluding the participants who used anti-hypertensive medicine.

| **Models** | **SBP** | **DBP** | **MAP** |
| --- | --- | --- | --- |
| Model 1 | 0.120(-0.005, 0.246) | 0.094(0.018, 0.169)* | 0.103(0.015, 0.190)* |
| Model 2 | 0.108(-0.019, 0.234) | 0.090(0.014, 0.166)* | 0.096(0.008, 0.183)* |
| Model 3 | 0.104(-0.023, 0.230) | 0.087(0.011, 0.163)* | 0.092(0.005, 0.180)* |
| Model 4 | 0.116(-0.011, 0.244) | 0.074(-0.003, 0.150) | 0.088(-0.001, 0.176) |
| Model 5 | 0.198(0.057, 0.340)* | 0.121(0.036, 0.206)* | 0.147(0.048, 0.245)* |

Data are *β* (95% CI). ^*^, *P* < 0.05.

SBP, systolic blood pressure; DBP, diastolic blood pressure; MAP, mean arterial pressure.

Model 1: adjusted for age, marital status, education level, family per capita yearly income, smoking, alcohol consumption, physical activity, adequate vegetables and fruits intake, high fat diet, family history of hypertension, and BMI; Model 2: adjusted as in model 1 plus age at menarche, menopause status, breast feeding, and use of oral contraceptive pills; Model 3: adjusted as in model 2 plus gestational hypertension and gestational diabetes mellitus; Model 4: adjusted as in model 3 plus parity; Model 5: adjusted as in model 4 plus age at last birth.

**Supplementary Table 7.** Sensitivities analyses of the association between age at first birth and hypertension, SBP, DBP, and MAP.

| **Models** | **Hypertension** | **SBP** | **DBP** | **MAP** |
| --- | --- | --- | --- | --- |
| Model 1 | 1.019(1.003, 1.036)* | 0.057(-0.063, 0.178) | 0.093(0.023, 0.164)* | 0.081(-0.001, 0.164) |
| Model 2 | 1.015(0.999, 1.032) | 0.042(-0.079, 0.163) | 0.086(0.015, 0.157)* | 0.071(-0.011, 0.154) |
| Model 3 | 1.015(0.998, 1.032) | 0.037(-0.084, 0.158) | 0.083(0.012, 0.154)* | 0.068(-0.015, 0.151) |
| Model 4 | 1.015(0.998, 1.032) | 0.055(-0.067, 0.178) | 0.069(-0.003, 0.140) | 0.064(-0.019, 0.148) |
| Model 5 | 1.023(1.004, 1.042)* | 0.147(0.010, 0.284)* | 0.123(0.043, 0.203)* | 0.131(0.038, 0.225)* |

Data are OR (95% CI) or *β* (95% CI). ^*^, *P* < 0.05.

Model 1: adjusted for age, marital status, education level, family per capita yearly income, smoking, alcohol consumption, physical activity, adequate vegetables and fruits intake, high fat diet, family history of hypertension, and BMI; Model 2: adjusted as in model 1 plus age at menarche, menopause status, breast feeding, and use of oral contraceptive pills; Model 3: adjusted as in model 2 plus gestational hypertension and gestational diabetes mellitus; Model 4: adjusted as in model 3 plus parity; Model 5: adjusted as in model 4 plus age at last birth.
