# Supplementary material for: Is first pregnancy age associated with hypertension in the Chinese rural women population?
Source: Front Public Health. 2023 Apr 17;11:1120732. doi: 10.3389/fpubh.2023.1120732 (PMC10150638; doi:10.3389/fpubh.2023.1120732)
Supplement: Supplementary file 2 [file Table_1.DOCX]

Hypertension is a principal cause of cardiovascular disease (CVD), which has become one of the most severe diseases in the world and China. Previous studies have elicited that those reproductive factors including age at menarche, age at menopause, parity, and gestational diabetes mellitus might impact future cardiovascular disease. However, the relationship between first pregnancy age and hypertension is uncertain, especially in resource-limited rural areas.

To our knowledge, this is the first description of the association of first pregnancy age with hypertension for women in rural areas of China, which provides a crucial piece of evidence for the rural Chinese population. The current results indicate that the later the first pregnancy age might increase the risk of hypertension. First pregnancy age might be an independent risk factor for hypertension in women.
